# Spinal cord compression in thoracolumbar burst fractures: application of high-definition three-dimensional exoscope in minimally invasive lateral surgery

**DOI:** 10.1007/s00590-022-03319-7

**Published:** 2022-07-26

**Authors:** Pietro Domenico Giorgi, Maria Ludovica Pallotta, Simona Legrenzi, Michele Nardi, Manzoni Andrea, Giuseppe Rosario Schirò

**Affiliations:** 1 Orthopedics and Traumatology Unit, Emergency and Urgency Department, A.S.S.T. Grande Ospedale Metropolitano Niguarda, Milan, Italy; 2 Orthopedics and Traumatology Residency, Università Degli Studi Di Milano, Milan, Italy; 3 Orthopedics and Traumatology Unit, Azienda Ospedaliera Universitaria Città Della Salute E Della Scienza Di Torino, Turin, Italy; 4 Neurosurgery Unit, Emergency and Urgency Department, A.S.S.T. Grande Ospedale Metropolitano Niguarda, Milan, Italy

**Keywords:** Exoscope, Spine, Spine surgery, Minimally invasive

## Abstract

**Study design:**

Spinal cord decompression in thoracolumbar burst fractures is challenging. Development of minimally invasive approaches and the improvement in new magnification technologies allowed a better and safer surgical treatment for these complex spinal injuries. We reported our experience in the minimally invasive surgical treatment of thoracolumbar burst fractures with spinal cord compression supported by high-definition (HD) three-dimensional (3D) Video-assisted telescope operating monitor (VITOM) or exoscope.

**Objectives:**

To assess the role and potential advantages of exoscope in the minimally invasive surgery of traumatic thoracolumbar spinal cord compression comparing traditional magnification systems.

**Setting:**

The study was conducted in a Northern Italy Spinal Trauma Center.

**Methods:**

We reported 10 consecutive thoracolumbar (T11-L2) burst fractures associated with spinal cord compression treated with minimally invasive corpectomy and exoscope-assisted spinal decompression. Three main indicators were retrospectively analyzed: surgical time, blood loss, and intraoperative complications. The data were compared with those obtained from an equal sample of 10 procedures performed by the same surgeon with the same technique, but traditional microscope assisted. User impressions in terms of ergonomics, magnification, and image quality were rated differently.

**Results:**

A small reduction of surgical time and blood loss were observed in the exoscope assisted group. There were no intraoperative complications attributed to visualization mode or conversion to the traditional microscope in any procedure. In our experience the exoscope allowed a better magnification and image definition with better ergonomics and user-friendliness.

**Conclusions:**

In our preliminary experience the exoscope new technology is a safe and effective tool for spinal cord minimally invasive decompression in thoracolumbar burst fractures. The stereoscopic vision provided by 3D images seems to be crucial in hand eye coordination. There are clear advantages in terms of maneuverability, wide field of view, deep focus, and more comfortable posture for the spinal surgeon.

## Introduction

Thoracolumbar burst fractures represent approximately 20% of all spine fractures and they can result in important neurological damage [1–3]. Nevertheless, their surgical management remains controversial [4, 5] especially regarding the treatment of spinal cord compression. In recent years, the minimally invasive approaches were developed in an effort to reduce the morbidity associated to the traditional open access [6, 7]. However, due to the small surgical field, a good visualization and illumination with unobstructed access is needed. During last decades, the magnification systems have exponentially upgraded as well as the surgeons’ confidence in performing microsurgical procedures. In particular, the application of video-assisted telescope operating monitor (VITOM) or exoscope is growing in popularity [8, 9]. We reported our preliminary experience in 10 cases of burst fractures with spinal cord compression treated with minimally invasive lateral surgery using a high-definition (HD) three-dimensional (3D) exoscope.

## Materials and methods

All cases were thoracolumbar (T11-L2) burst fractures (A3 or A4 according to the AOSpine Classification System [10] associated with spinal cord compression with complete or incomplete neurological damage. All procedures of minimally invasive corpectomy with spinal decompression exoscope assisted were performed by a single highly experienced spinal orthopedic surgeon. Three main indicators were analyzed: surgical time, blood loss, and intraoperative complications. Moreover, impressions in terms of ergonomics, preparation, magnification, image definition, illumination, and user-friendliness were rated differently by the surgeon and assistant with questionnaires with five-level Likert items (from 5 = very good to 1 = very insufficient) regarding preparation (including setup, balancing, positioning, and draping), image definition, magnification, illumination, ergonomics, and user-friendliness (including using zoom and focus, changing the position of the visual field). We performed separate analyses for the surgeon and assistant to assess differences in perception as well as a pooled analysis to give median scores for overall comparison.

These data were retrospectively compared with those obtained from an equal sample of 10 procedures performed by the same surgeon with the same technique, but traditional microscope assisted. Local ethical committee approval was not required as patients were treated according to generally accepted standards of care. Moreover, the exoscope was used as a fully approved medical device without research intention.

### High-definition three-dimensional exoscope

**Fig. 1:**
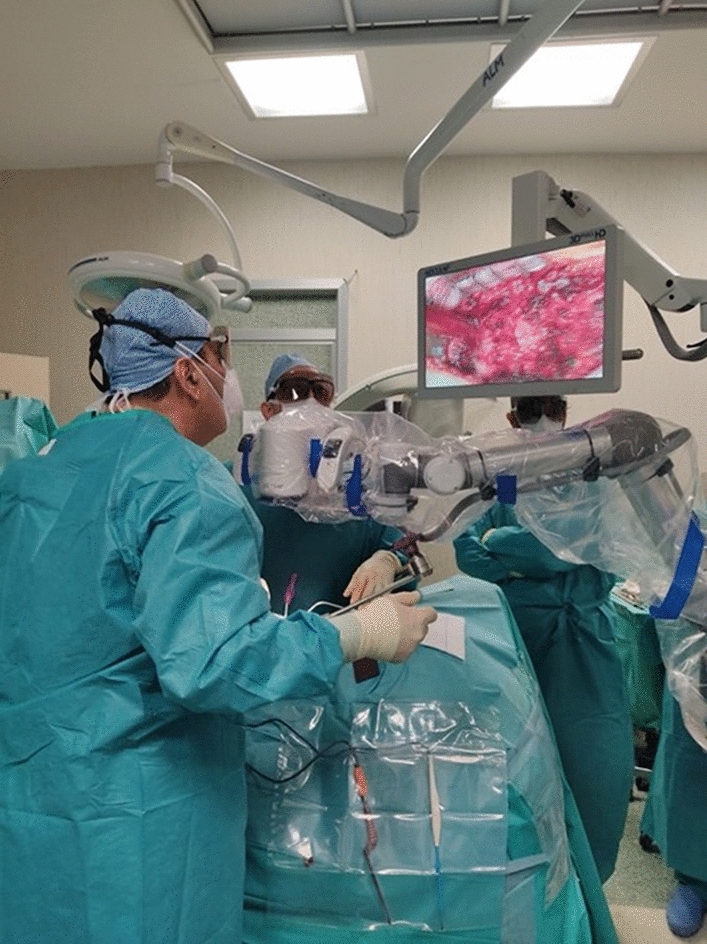
Operating setting with the robotic arm positioned on the surgical field and the surgeon performs the spinal cord decompression looking at the 32’’ 3D monitor

**Fig. 2:**
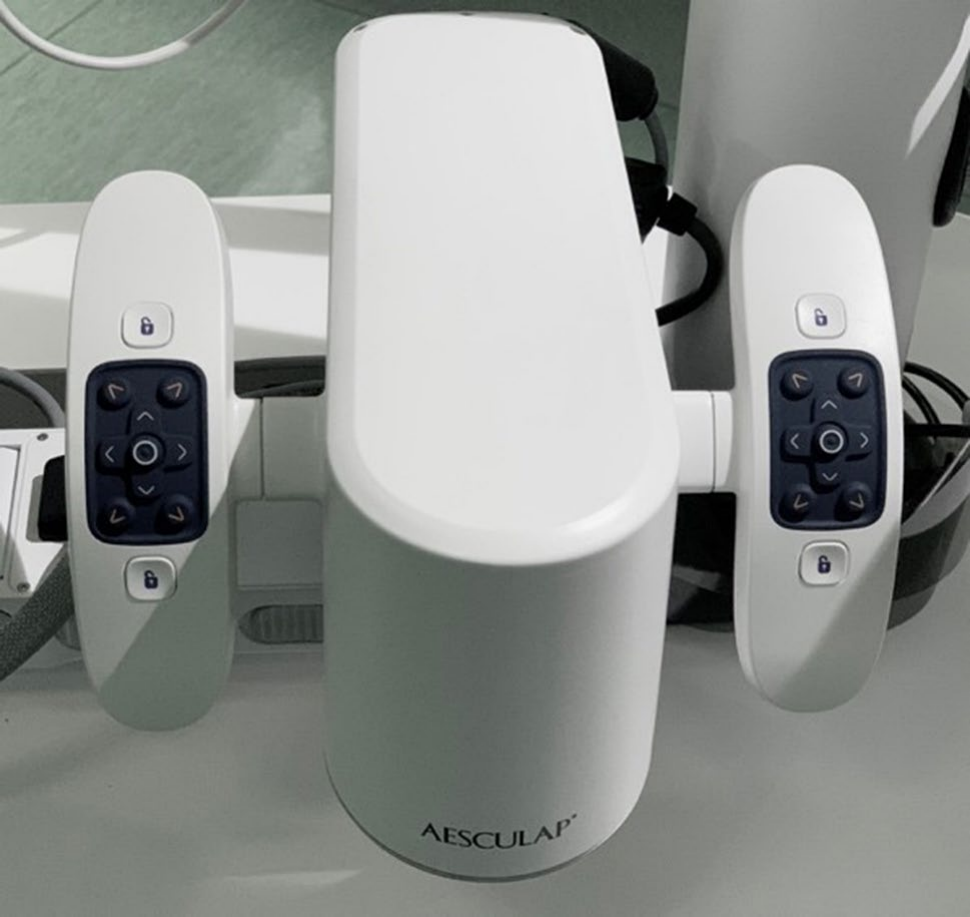
Two different control areas are located on the right and the left side. The surgeon can choose the position of the commands in the 2 areas in order to move the robotic arm, to select the light intensity and to check the focus. Moreover, it is possible to take pictures and videos and save the position of the arm in the space

Exoscopy developed and refined over the past decades, evolving from former standard definition cameras and 2D visualization systems toward HD cameras and 3D instrumentations, allowing a wide and effective application of a new technology called exoscope. It is a particular type of telescope with zoom (10x), automatic focus functions, integrated illumination, and robotic arm (Fig. 1). Every type of spinal surgery has a standardized operating setting. Through a control unit, the surgeon is able to focus, modify the magnification, shift the orientation of the small camera, and save the arm position in the space (Fig. 2). The surgical field is displayed on two movable 3D monitors (32’’ and 55’’) positioned on the opposite side of the surgical table in front of surgeon and the assistant. In order to obtain a comfortable visualization, the 32’’ and 55’’ screens should be positioned, respectively, 1.5 and 2 m away from surgeon. Images displayed on the 2 screens are specular to each other to reproduce the intraoperative sight with the correct orientation for each surgeon. Advantages of exoscope are also related to its longer focal distance and wider field of view. A 4 K exoscope with a focal distance of 200 mm to 500 mm was used. A 20–25 cm space between the camera and the surgical field allows the best clear visualization.

### Surgical technique

**Fig. 3:**
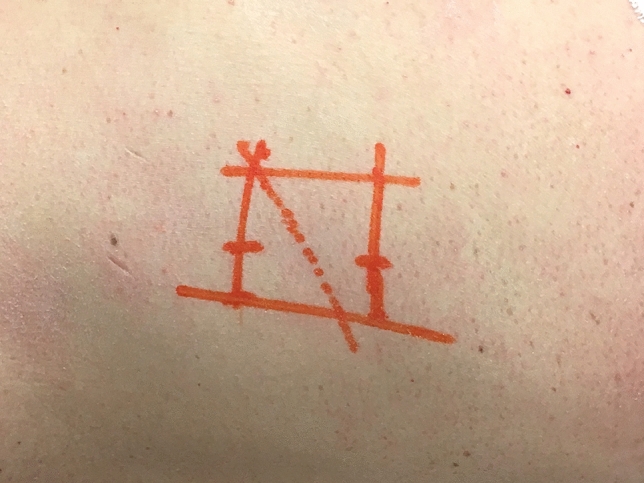
The skin incision (dashed line) is an oblique 6 cm incision on the left side of the thoracolumbar spine. The skin landmarks are the two endplates, the anterior and posterior wall of the vertebra (continuous lines)

The patients were placed in right lateral decubitus position, under general anesthesia. A minimally invasive lateral extracoelomic approach was used to expose the thoracolumbar junction. A 6-cm-long oblique incision was made following the trajectory of the rib at the index level, drawing specific skin landmarks under radioscopic guidance (Fig. 3). To facilitate the surgical approach, a portion of rib was removed. The parietal pleura was exposed, and the surgeon developed the space between the endothoracic fascia and the pleura. The posterior attachments of the diaphragm were sharply transected off the transverse process of L1 [11].

**Fig. 4:**
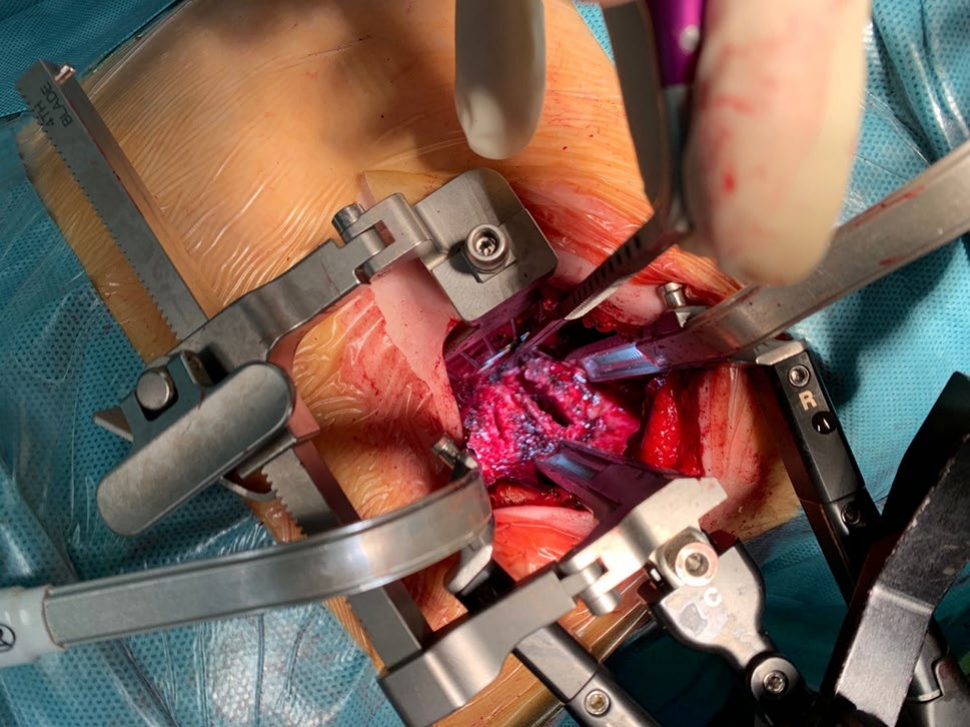
The surgical exposure through the tubular retractor placed in the retro cavitary space

**Fig. 5:**
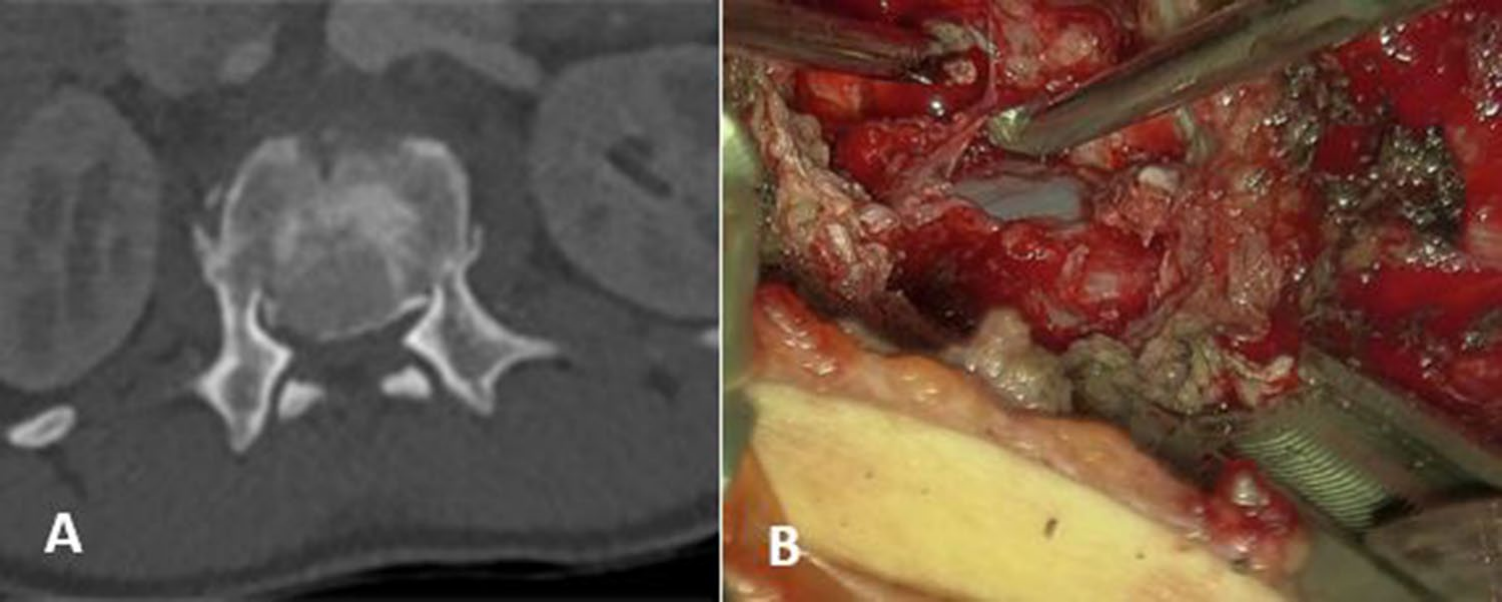
**A** A preoperative axial TC image shows the relevant endocanalar compression of a L1 burst fracture. **B** An intra operative image of the surgical field after complete corpectomy e decompression of the dural sac

Sequential tubular dilators were then inserted at the correct level, under fluoroscopic guidance. After the complete exposure of our target (the vertebral body and the two adjacent discs), a deep three-blade retractor was inserted and fixed to the operating table (Fig. 4). The exoscope was then introduced in the operating setting and maintained over the surgical field for the rest of the procedure. The adjacent disks were removed and the corpectomy was performed with diamond cutter in order to minimize bleeding and to avoid the traumatic effect of osteotomes on the endocanalar structures. The pedicle was exposed and resected using the drill and the Kerrison. The epidural space was gently dissected from the retropulsed bone fragments that were completely removed (Fig. 5). Instrumentation included, scissors, bone nibblers, straight or curved curettes and, bone rasps. The final stage of posterior corporectomy should not be anteroposterior but on the contrary should push the intracanal bone fragments forward, using a curved curette. Posterior longitudinal ligament (PLL) exeresis enables visualization of the dura mater. Epidural bleeding was easily controlled with hemostats and full decompression was confirmed by reexpansion of the dural sac anteriorly. In case of traumatic durotomy a dural patch was applied according the triple-layer technique.

During the decompression step, the HD 3D exoscope showed substantial advantages compared to traditional microscope considering hits longer focal distance that allowed the positioning of the small camera farther from the surgical field, providing an unobstructed working space and an easy introduction of surgical instruments. Moreover, the comfortable ergonomic position and the possibility to shift from microscopic to macroscopic vision in a rapid and easy way thanks to the robotic arm speeded up the procedure (Table 1).

**Table 1:** Comparison of microscope and exoscope

	Ergonomics		Preparation		Magnification		Illumination		Definition		User-friendliness	
	S	A	S	A	S	A	S	A	S	A	S	A
Exoscope	5	5	3	4	5	5	4	5	5	5	5	5
Microscope	2	3	3	3	4	4	4	4	4	4	3	3

Results of category ratings by surgeon (S) and assistant (A) on a five-level scale (1 = very insufficient, 2 = insufficient, 3 = neutral, 4 = good, 5 = very good)

Finally, a titanium expandable cage filled by the autologous bone (the removed rib) was inserted in the intervertebral space and expanded under radioscopic control. After careful hemostasis and removal of the deep retractor, the integrity of the pleura was checked. Fascia and skin closure were than completed.

## Results

Over a period of six months, from July 2021 to December 2021, a total of 10 consecutive minimally invasive lateral thoracolumbar surgeries were performed using an HD 3D exoscope in the orthopedic department of our hospital, in Milan. Demographic data of the 2 groups are described in Table 2.

**Table 2:** Demographic data of the 2 groups compared

	Exoscope group	Microscope group
Number of patients	10	10
Mean age (y.o.)	42.7	45.9
Male	4	5
Female	6	5
BMI (average)	24.1	23.4

In the exoscope group 7 cases of A4 type and 3 cases of A3 type fractures were recorded, while in the microscope group there were 6 cases of A4 type and 4 cases of A3 type fracture.

None of the procedures had to be stopped because of technical problems. There were no intraoperative complications attributed to the visualization mode or conversion to the traditional microscope in any procedure. In the exoscope group a reduction of the surgical time and blood loss was recorded with a mean value of 155 min (87–193 min) and 403 cc (202–510 cc). In the comparison group the mean surgical time was 177 min (107–198 min) and the mean blood loss was 421 cc (151–563 cc). In surgeon experience the exoscope required a shorter learning curve compared to microscopy and allowed a better ergonomics, magnification, definition, and user-friendliness. However, the preparation and illumination resulted comparable (Table 1).

## Discussion

Spinal cord decompression in thoracolumbar burst fracture is a challenge in spinal surgery. During last decades the development of minimally invasive approaches and the improvement of magnification technologies allowed a better and safer surgical treatment for these complex injuries. Telescopic systems consist of cameras with high magnification, a mobile mechanical arm and projecting screens. Recently, this type of technology upgraded from the 2D visualization systems toward the HD cameras and 3D instrumentations, allowing a wide and effective application in different surgical specialties [8, 9, 12–14]. Rossini et al. reported their preliminary satisfactory results in the treatment of cranial tumor supported by a 3D exoscope. Also in spinal surgery many applications of exoscope have been reported [14]. But, to the best of our knowledge, this is the first report concerning spinal cord decompression with VITOM 3D as a visualization instrument in minimally invasive lateral spinal surgery. The small and deep surgical field of this approach is a challenging setting for the use of the magnification instruments. The traditional microscope is associated with some limitations mainly due to its large size obstructing the access of the surgical instruments. Also the use of endoscopic-assisted visualization has difficulties related to the lack of 3D visualization and poor image quality [8, 13]. In our experience, there are substantial advantages in the utilization the HD 3D exoscope for spinal decompression. It has a longer focal distance and wider field of view allowing the positioning of the small camera farther from the surgical field, providing an unobstructed working space and an easy introduction of surgical instruments. However, the illumination and depth are at least equal to the operating microscopy. In such a deep surgical field, the user-friendliness and the stereoscopic vision provided by 3D images seems to be crucial in hand eye coordination. Both surgeon and his assistant can work in a natural and comfortable ergonomic position. Moreover, it is possible to shift from microscopic to macroscopic vision in a rapid and easy way, without moving the exoscope or completely losing microscopic vision. According to Beez et al. [9] that reported a brief learning curve in pediatric neurosurgery, the adaptation to the exoscope was quite easy in our experience.

According to this preliminary study, the exoscope is a safe and effective tool for spinal cord minimally invasive decompression in thoracolumbar burst fractures. In such a small and deep surgical field, the stereoscopic vision provided by 3D images seems to be crucial in hand eye coordination. In authors’ preliminary experience, it has clear advantages, compared to the traditional microscope due to its maneuverability, wide field of view, deep focus, and more comfortable posture for the surgeons. This report registered a reduction in surgical time and blood loss. Nevertheless, more studies with bigger samples are needed to confirm the role of exoscope in spinal cord decompression.

## Data Availability

The data that support the findings of this study are available from the corresponding author upon reasonable request.
